# Insights into the segmental identity of post-oral commissures and pharyngeal nerves in Onychophora based on retrograde fills

**DOI:** 10.1186/s12868-015-0191-1

**Published:** 2015-08-25

**Authors:** Christine Martin, Georg Mayer

**Affiliations:** Animal Evolution and Development, Institute of Biology, University of Leipzig, Talstraße 33, 04103 Leipzig, Germany; Department of Zoology, Institute of Biology, University of Kassel, Heinrich-Plett-Str. 40, 34132 Kassel, Germany

**Keywords:** Central nervous system, Brain, Pharynx, Neuronal tracing, Arthropod, Panarthropod

## Abstract

**Background:**

While the tripartite brain of arthropods is believed to have evolved by a fusion of initially separate ganglia, the evolutionary origin of the bipartite brain of onychophorans—one of the closest arthropod relatives—remains obscure. Clarifying the segmental identity of post-oral commissures and pharyngeal nerves might provide useful insights into the evolution of the onychophoran brain. We therefore performed retrograde fills of these commissures and nerves in the onychophoran *Euperipatoides rowelli*.

**Results:**

Our fills of the anterior and posterior pharyngeal nerves revealed groups of somata that are mainly associated with the deutocerebrum. This resembles the innervation pattern of other feeding structures in Onychophora, including the jaws and several lip papillae surrounding the mouth. Our fills of post-oral commissures in *E. rowelli* revealed a graded arrangement of anteriorly shifted somata associated with post-oral commissures #1 to #5. The number of deutocerebral somata associated with each commissure decreases posteriorly, i.e., commissure #1 shows the highest and commissure #5 the lowest numbers of associated somata, whereas none of the subsequent median commissures, beginning with commissure #6, shows somata located in the deutocerebrum.

**Conclusions:**

Based on the graded and shifted arrangement of somata associated with the anteriormost post-oral commissures, we suggest that the onychophoran brain, which is a bipartite syncerebrum, might have evolved by a successive anterior/anterodorsal migration of neurons towards the protocerebrum in the last onychophoran ancestor. This implies that the composite brain of onychophorans and the compound brain of arthropods might have independent evolutionary origins, as in contrast to arthropods the onychophoran syncerebrum is unlikely to have evolved by a fusion of initially separate ganglia.

**Electronic supplementary material:**

The online version of this article (doi:10.1186/s12868-015-0191-1) contains supplementary material, which is available to authorized users.

## Background

**Fig. 1:**
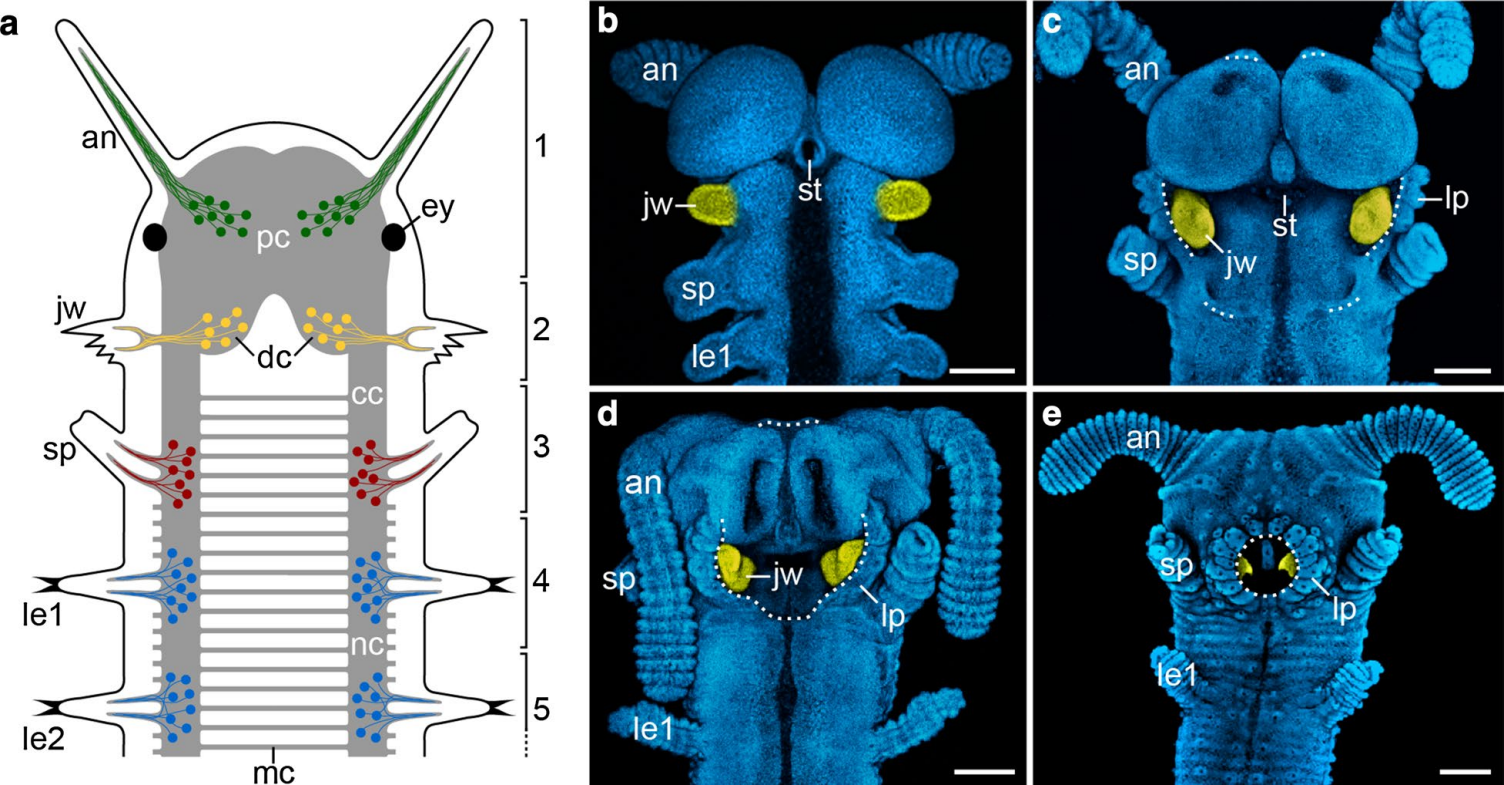
Segmental identity of cephalic appendages and head development in onychophorans. **a** Simplified diagram of the innervation of segmental cephalic appendages. Body segments, each carrying a pair of appendages, are numbered. **b**–**e** Head development in embryos of subsequent developmental stages in the onychophoran *Euperipatoides rowelli*. The jaws are highlighted artificially in *yellow*. Modified from Ou et al. [15]. *an* antenna, *cc* connecting cord, *dc* deutocerebrum, *ey* eye, *jw* jaw, *le1 and le2* first and second legs, *lp* lip papillae, *mc* median commissure, *nc* nerve cord, *pc* protocerebrum, *st* stomodeum, *sp* slime papilla. *Scale bars* (in **b**–**e**) 200 µm

The typical arthropod brain is composed of three segmental regions: the protocerebrum, deutocerebrum, and tritocerebrum [1–6]. In contrast, the brain of one of the closest arthropod relatives, the Onychophora (velvet worms), contains only two segmental regions corresponding to the arthropod proto- and deutocerebrum [7, 8]. Alternative hypotheses suppose a tripartite onychophoran brain [9] but are based on a misinterpretation of the onychophoran neuroanatomy, as the authors mistook the jaw nerve for the slime papilla nerve. Furthermore, a recent study [10] proposes that the onychophoran brain is a monopartite structure based on *engrailed* mRNA expression [11, 12], a method that is not suitable for addressing brain segmentation, as the anterior *engrailed* stripe is on the non-neuroectodermal side [13]. Despite the lack of a distinct border delineating these two brain regions, the protocerebrum of onychophorans contains the central body and the mushroom bodies and innervates the antennae and eyes, whereas the deutocerebrum is defined by the position of neuronal somata supplying the appendages of the second body segment, i.e., the jaws [7, 8, 14] (Fig. 1a). The onychophoran jaws are the only appendages that become incorporated into the definitive mouth cavity during embryonic development [14, 15] (Fig. 1b–e). In contrast to the antennae and jaws, the third pair of cephalic appendages of onychophorans, the slime papillae, is not innervated by the brain but rather by the anterior portions of the ventral nerve cords [7, 8, 16] (Fig. 1a).

The two widely separated ventral nerve cords of onychophorans do not show any segmental ganglia but instead are linked with each other by numerous median and ring commissures in an orthogon-like fashion [16–19]. The nerve cords are connected to the brain via a pair of medullary (i.e. non-ganglionated) structures, the so-called connecting cords [7] (“circumoesophageal connectives” sensu Henry [20]; “medullary connectives” sensu Whitington and Mayer [16] “connecting pieces” sensu Martin and Mayer [14]). The frequently used term “circumoesophageal connectives” is unsuitable for designating these cords, as connectives are somata-free structures connecting ganglia [5], whereas the connecting cords resemble the ventral nerve cords. Nevertheless, the connecting cords differ from the ventral nerve cords in that they are not associated with ring commissures. The connecting cords are situated on each side of the pharynx and linked with each other via a number of post-oral (=post-pharyngeal) commissures. Due to the medullary organization of the connecting cords, their segmental identity as well as that of the associated commissures is unclear.

In addition to the post-oral commissures, Henry [20] described a pair of “stomodaeal nerves” emanating from the connecting cords and supplying the pharynx. As with the post-oral commissures associated with the connecting cords, the segmental identity of this pair of pharyngeal nerves is unknown because the corresponding neuronal somata have not been identified. An additional prominent “loop nerve” is also associated with the onychophoran pharynx [4]. Although this nerve shows a medullary organization, with at least some somata located in the dorsolateral pharyngeal wall, it is unclear whether or not there are additional neurons supplying this nerve located within the brain. Clarifying this issue would help to determine the innervation pattern and segmental identity of the onychophoran pharynx, which remains ambiguous.

During ontogeny, the onychophoran pharynx arises from the walls of the embryonic stomodeum [15, 21]. This would suggest that the pharynx, like the stomodeum itself, belongs to the second body segment. Accordingly, the first post-oral commissure of onychophorans, which is closely associated with the ventral pharyngeal wall [20], might also belong to the second body segment. Alternatively, this commissure might be composed of fibers from both the second (jaw) and the third (slime papilla) segments, thus resembling the situation in arthropods, in which the first post-oral commissure contains both deutocerebral and tritocerebral fibers [1, 22–26].

To clarify the composition of post-oral commissures and the segmental identity of pharyngeal nerves in Onychophora, we performed retrograde fills of these neural structures and localized the position of their supplying neurons in the onychophoran *Euperipatoides rowelli*.

## Results

### Arrangement and innervation pattern of the anterior post-oral commissures

**Fig. 2:**
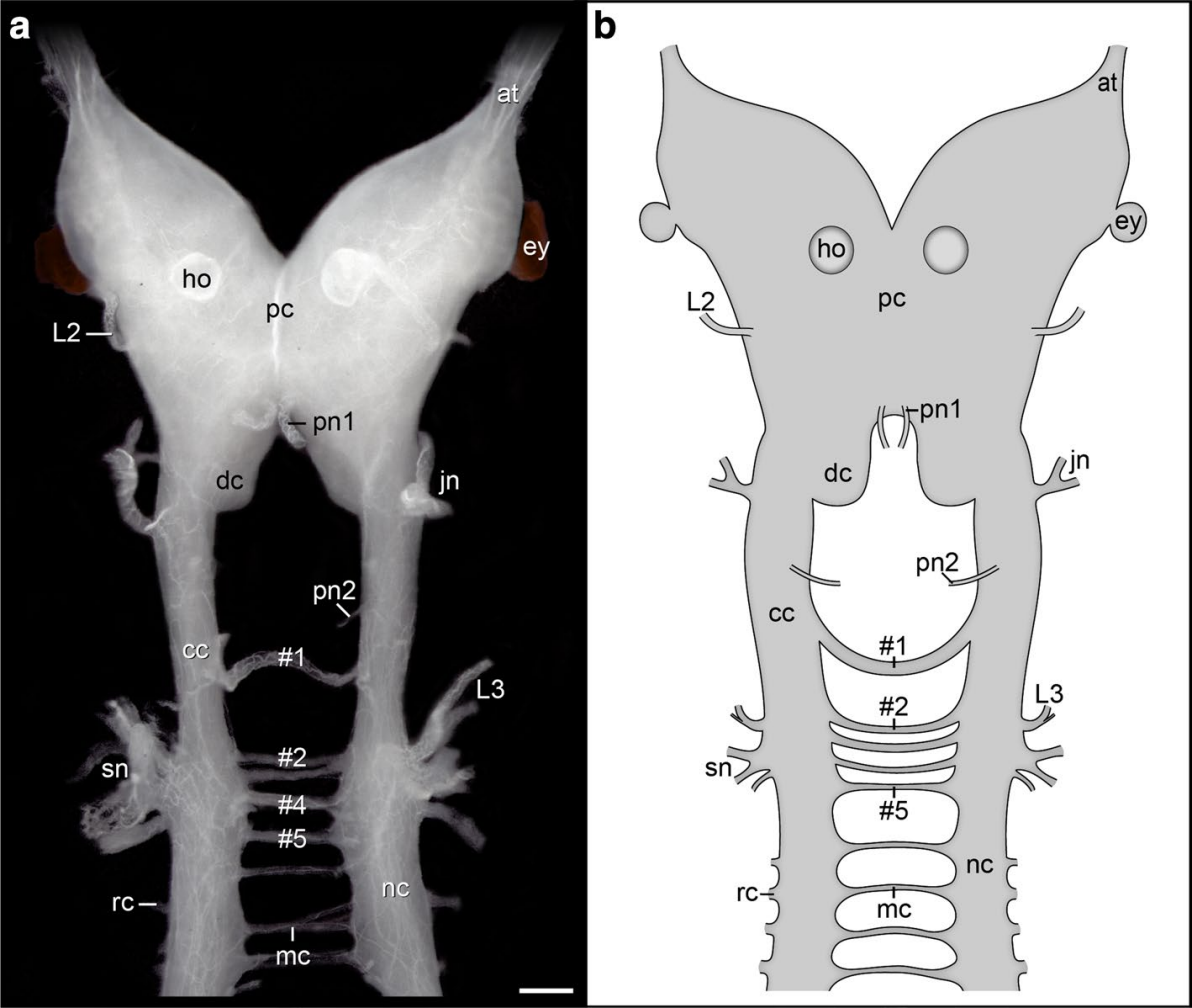
Anatomy of the anterior nervous system in the onychophoran *Euperipatoides rowelli*. Light micrograph (**a**) and simplified diagram (**b**) of dissected brain and anterior nerve cords in ventral view. Anterior is *up*. Anteriormost post-oral commissures numbered (#1 to #5). *at* antennal tract, *cc* connecting cord (characterized by the absence of ring commissures), *dc* deutocerebrum, *ey* eye, *ho* hypocerebral organ, *jn* jaw nerve, *L2 and L3* second and third lip papillae nerves; mc, median commissure, *nc* nerve cord, *pc* protocerebrum, *pn1 and pn2* anterior and posterior pharyngeal nerves, *rc* ring commissure, *sn* slime papilla nerves. *Scale bar* (in **a**) 200 µm

Dissected portions of the anterior nervous system of *E. rowelli* show that the arrangement of the five anteriormost median commissures differs from that of the remaining commissures connecting the two nerve cords of the trunk (Fig. 2a, b). The first commissure links the two ring commissure-free connecting cords at a distance of about one-third between the slime papilla and the jaw nerves. This prominent commissure is longer and thicker than the remaining post-oral commissures, from which it is segregated by a wider gap. The first post-oral commissure forms a ventral loop around the pharynx and is attached to the ventral pharyngeal wall by thin tissue fibers so that this commissure usually has to be dissected from the pharynx during preparation. The commissures #2 to #5 link the nerve cords at the level of the third lip papillae nerve and the slime papillae nerves (Fig. 2a, b). These commissures lie closer to each other than do the remaining median commissures of the trunk.

**Fig. 3:**
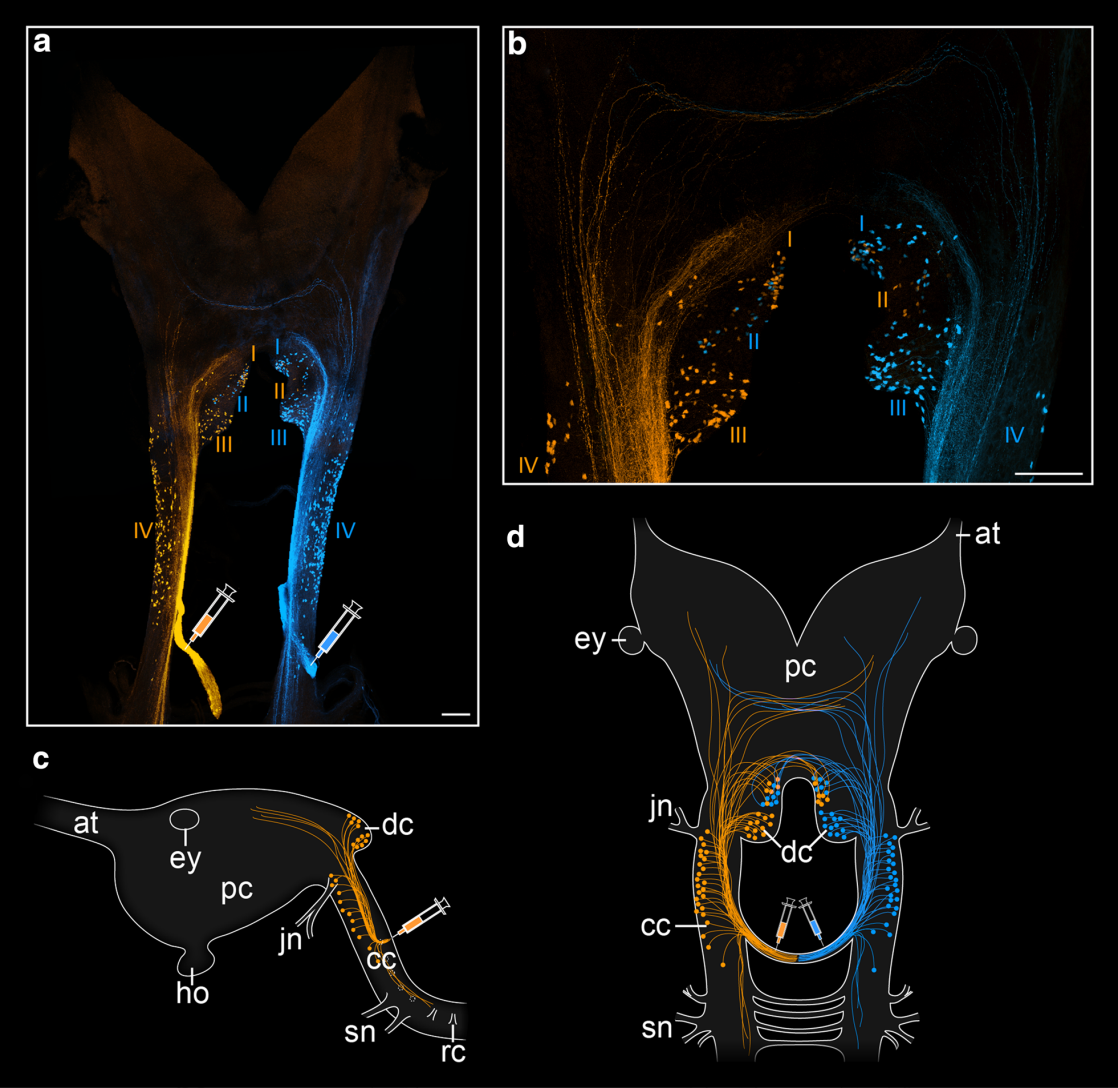
Localization of neuronal somata associated with the first post-oral commissure in *Euperipatoides rowelli.* Retrograde fills. *Syringes* indicate the corresponding fill sites. **a**, **b** Maximum projection confocal micrograph of a double fill with dextran coupled to two different fluorochromes. Dorsal view, anterior is *up*. Note the position of somata in the connecting cords (group IV) and in the deutocerebrum (groups I–III), some of which lie in the contralateral brain hemisphere (group II). **c** Overview diagram illustrating the position of neurons associated with the first post-oral commissure in lateral view. **d** Overview diagram demonstrating the position of neurons associated with the first post-oral commissure in dorsal view. *at* antennal tract, *cc* connecting cord, *dc* deutocerebrum, *ey* eye, *ho* hypocerebral organ, *jn* jaw nerve, *pc* protocerebrum, *rc* ring commissure, *sn* slime papilla nerves. *Scale bars* (in **a**, **b**) 100 µm

To determine the position of neuronal somata associated with the anterior post-oral commissures, we performed retrograde fills of commissures #1 to #5 and compared their innervation pattern to that of the median commissures from the remaining trunk. Our bidirectional fills revealed eight groups of somata associated with the first post-oral commissure, i.e., four groups labeled by each fill (Fig. 3a–d). Three of the four groups are located dorsally within the deutocerebrum: two (with ~30 and ~50 somata each) in the ipsilateral half and one (with ~20 somata) in the contralateral half of the deutocerebrum, with respect to the site of the fill. The fourth and largest group of ~100 somata is located laterally in each connecting cord. The somata of this group are distributed along each connecting cord from the basis of the first post-oral commissure to the basis of the jaw nerve (Fig. 3a, c, d). In addition to the neuronal somata, our fills of the first post-oral commissure revealed numerous anterior and posterior fibers, which terminate within both the protocerebrum and the nerve cords (Fig. 3a–d).

**Fig. 4:**
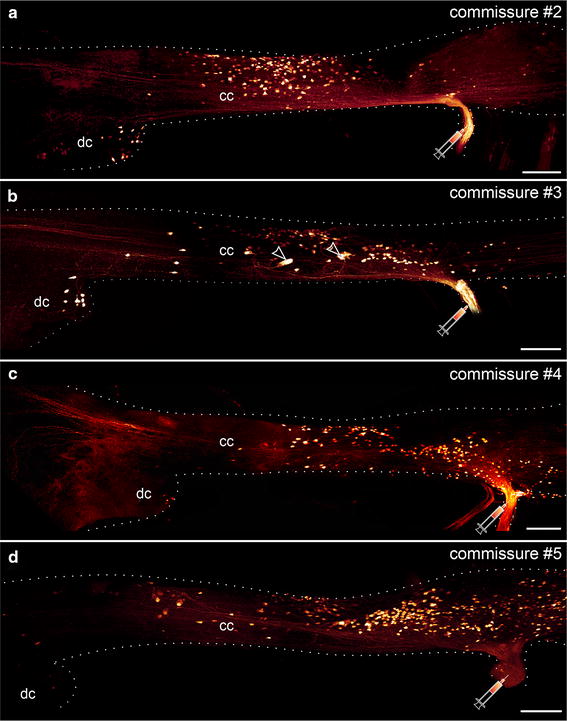
Localization of neuronal somata associated with the second to fifth post-oral commissures in *Euperipatoides rowelli.* Maximum projection confocal micrographs of the right portion of the anterior nervous system in dorsal view with the corresponding commissures filled. Anterior is *left* in all images. *Syringes* indicate the corresponding fill sites. Note that most labeled somata are situated in the connecting cord and only a few are located within the deutocerebrum. Note also that both the number of deutocerebral somata and the anterior shift in position of somata within the connecting cord decrease from anterior (commissure #2) to posterior (commissure #5). *Arrowheads* (in **b**) point to relatively large somata associated with the third post-oral commissure. *cc* connecting cords, *dc* deutocerebrum. *Scale bars* 100 µm

In contrast to the first post-oral commissure, commissures #2 to #5 do not show any contralateral clusters of somata within the brain with respect to the site of the fill (Fig. 4a–d). All somata are instead located in the ipsilateral half of the nervous system, including the deutocerebrum and the connecting cord. The number of somata located within the deutocerebrum decreases from commissure #2 to commissure #5. The somata associated with each commissure exhibit an anterior shift in the connecting cord with respect to the basis of the corresponding commissure. This shift is more prominent for the anteriormost commissures and less evident for those situated further posteriorly (Figs. 3a, 4a–d). In contrast to the first post-oral commissure, the somata associated with commissures #2 to #5 are not located laterally in the connecting cord but instead occupy most of its width. Most filled somata associated with post-oral commissures #2 to #5 are uniform in size (5–10 µm in diameter), but a few relatively large somata (25–30 µm in diameter) are associated with commissure #3 (arrowheads in Fig. 4b). Like the first post-oral commissure, commissures #2 to #5 exhibit additional anterior fibers that project into the protocerebrum.

**Fig. 5:**
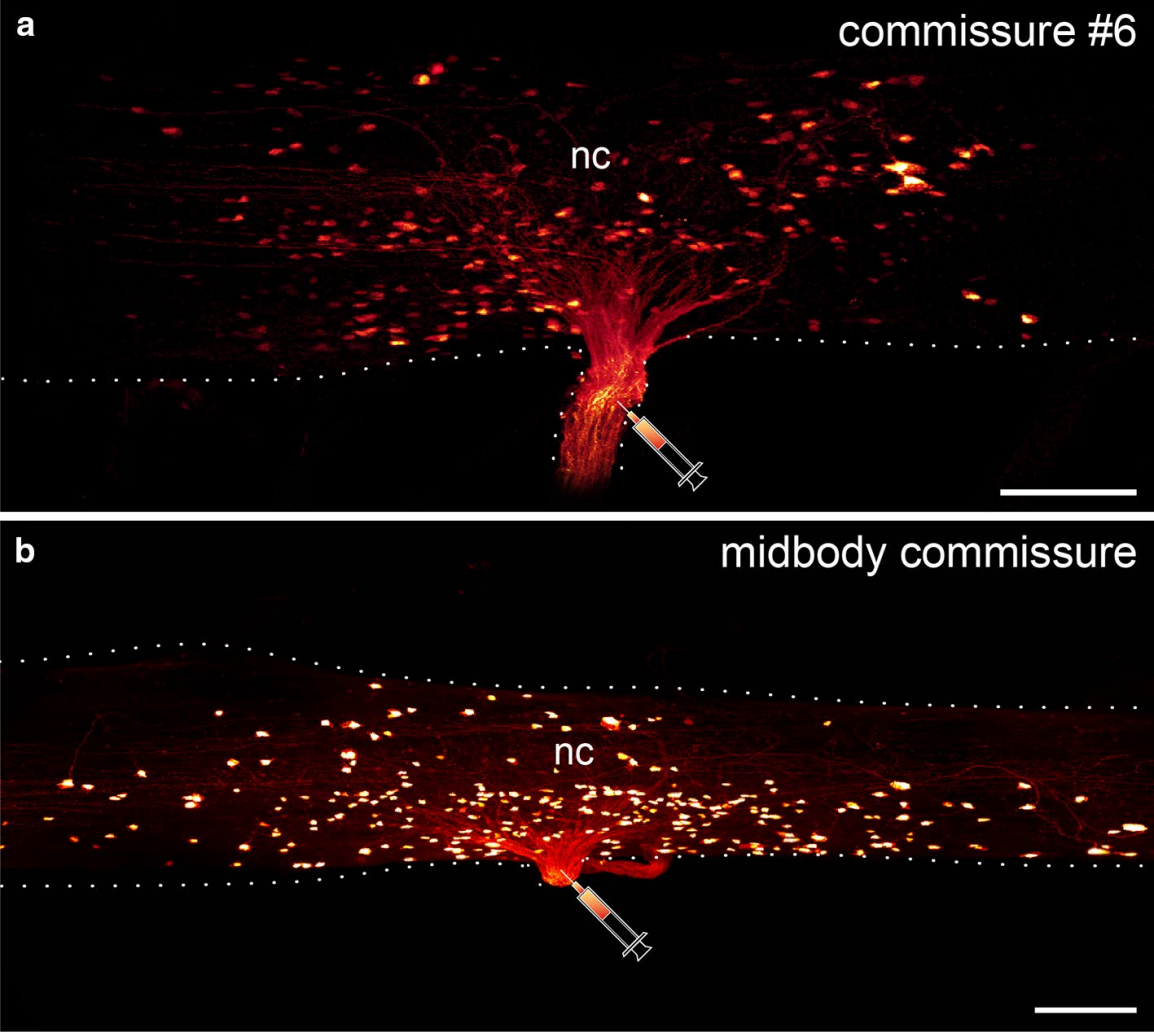
Localization of neuronal somata associated with the sixth post-oral and a midbody commissure in *Euperipatoides rowelli.* Retrograde fills. **a**, **b** Maximum projection confocal micrographs. Anterior is *left* in both images. *Syringes* indicate the corresponding fill sites. Note the similar arrangement of neuronal somata that are grouped near the basis of each commissure and do not show any anterior shift in position. *nc* nerve cord. *Scale bars* 100 µm

While a slight anterior shift in the position of somata associated with commissures #2 to #5 is evident within the connecting cords, the remaining median commissures of the trunk, beginning with commissure #6, do not show any such shift. All somata associated with these commissures are instead grouped at the level of each commissure (Fig. 5a, b).

### Neuronal tracing of nerves supplying the pharynx

**Fig. 6:**
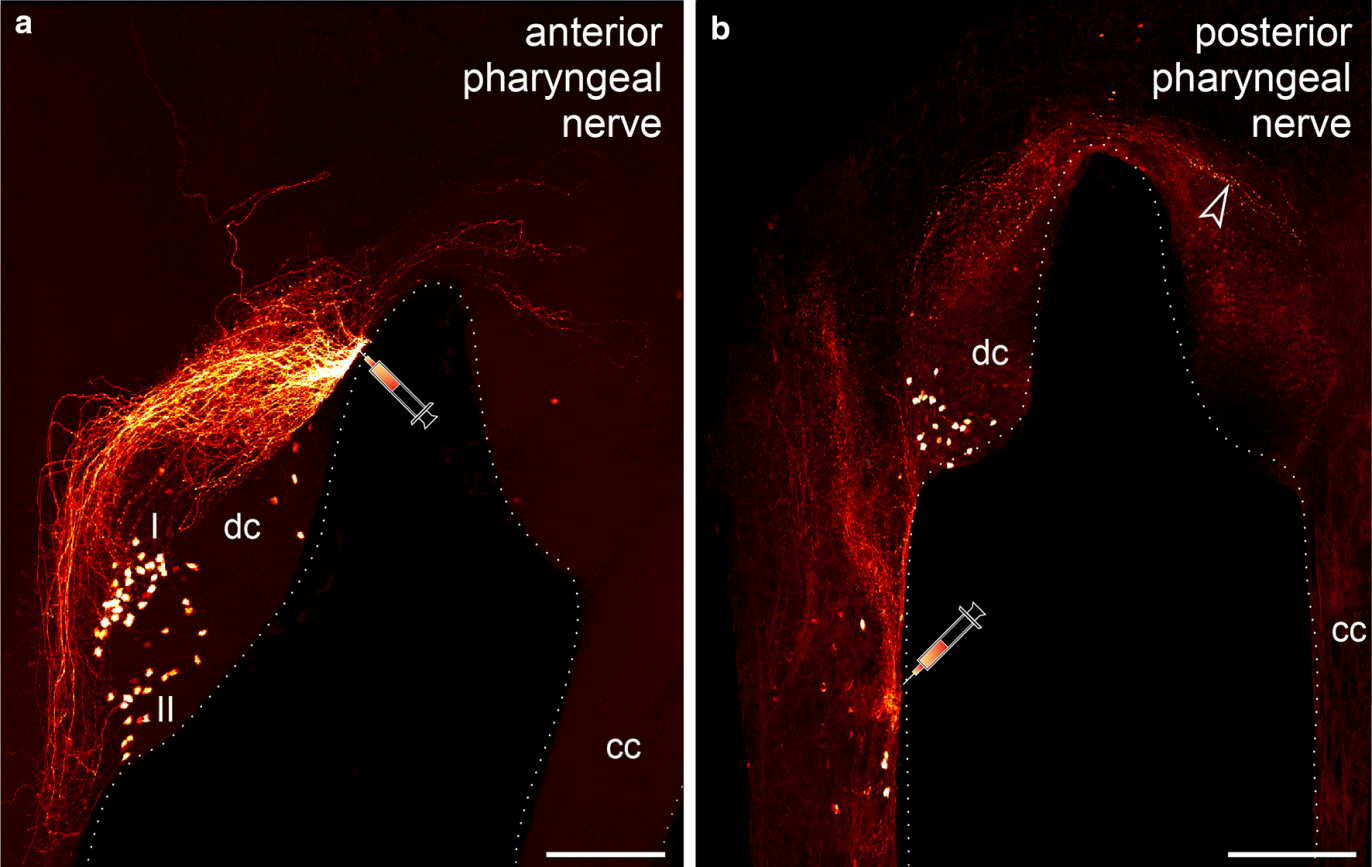
Localization of neuronal somata associated with the anterior and posterior pharyngeal nerves in *Euperipatoides rowelli.* Retrograde fills. Maximum projection confocal micrographs in dorsal view. Anterior is *up*. *Syringes* indicate the corresponding fill sites. **a** Fill of the first pharyngeal nerve showing two groups of somata (I and II) associated with the deutocerebrum. **b** Fill of the second pharyngeal nerve illustrating neuronal somata within the deutocerebrum as well as in the connecting cord. *Arrowhead* points to fibers terminating contralaterally. *cc* connecting cord, *dc* deutocerebrum. *Scale bars* 100 µm

Of the two pairs of nerves supplying the pharynx in *E. rowelli*, the anterior pair enters the brain ventrally, where it is associated with the anterior portion of the deutocerebrum (Figs. 2a, b, 6a). In contrast, the posterior pair of pharyngeal nerves is associated with the portions of the connecting cords anterior to the first post-oral commissure (Figs. 2a, b, 6b). Retrograde fills of the anterior pharyngeal nerve revealed ~60 somata located exclusively within the deutocerebrum in the same brain hemisphere, posterior to the basis of this nerve (Fig. 6a). There are two major groups of somata: those of group I (Fig. 6a) project their axons exclusively anteriorly, whereas the axons of group II (Fig. 6a) leave the somata posteriorly and are then reoriented anteriorly. In addition, there are a few somata that are located further anteriorly within the deutocerebrum (Fig. 6a). Some of the fibers associated with the anterior pharyngeal nerve extend further anteriorly in the protocerebrum as well as in the contralateral brain hemisphere, where they terminate.

Like the anterior pair of nerves supplying the pharynx, the posterior pair also shows neuronal somata located within the deutocerebrum (Fig. 6b). However, apart from these, each posterior pharyngeal nerve has a few additional somata (~10) in each connecting cord that are grouped around the basis of the nerve (Fig. 6b). The group of somata within the deutocerebrum comprises ~30 cells that are all situated in the ipsilateral brain hemisphere. A number of fibers associated with the posterior pharyngeal nerve terminate in the connecting cord as well as in the contralateral half of the brain (arrowhead in Fig. 6b).

## Discussion

### Deutocerebral innervation of feeding structures in Onychophora

**Fig. 7:**
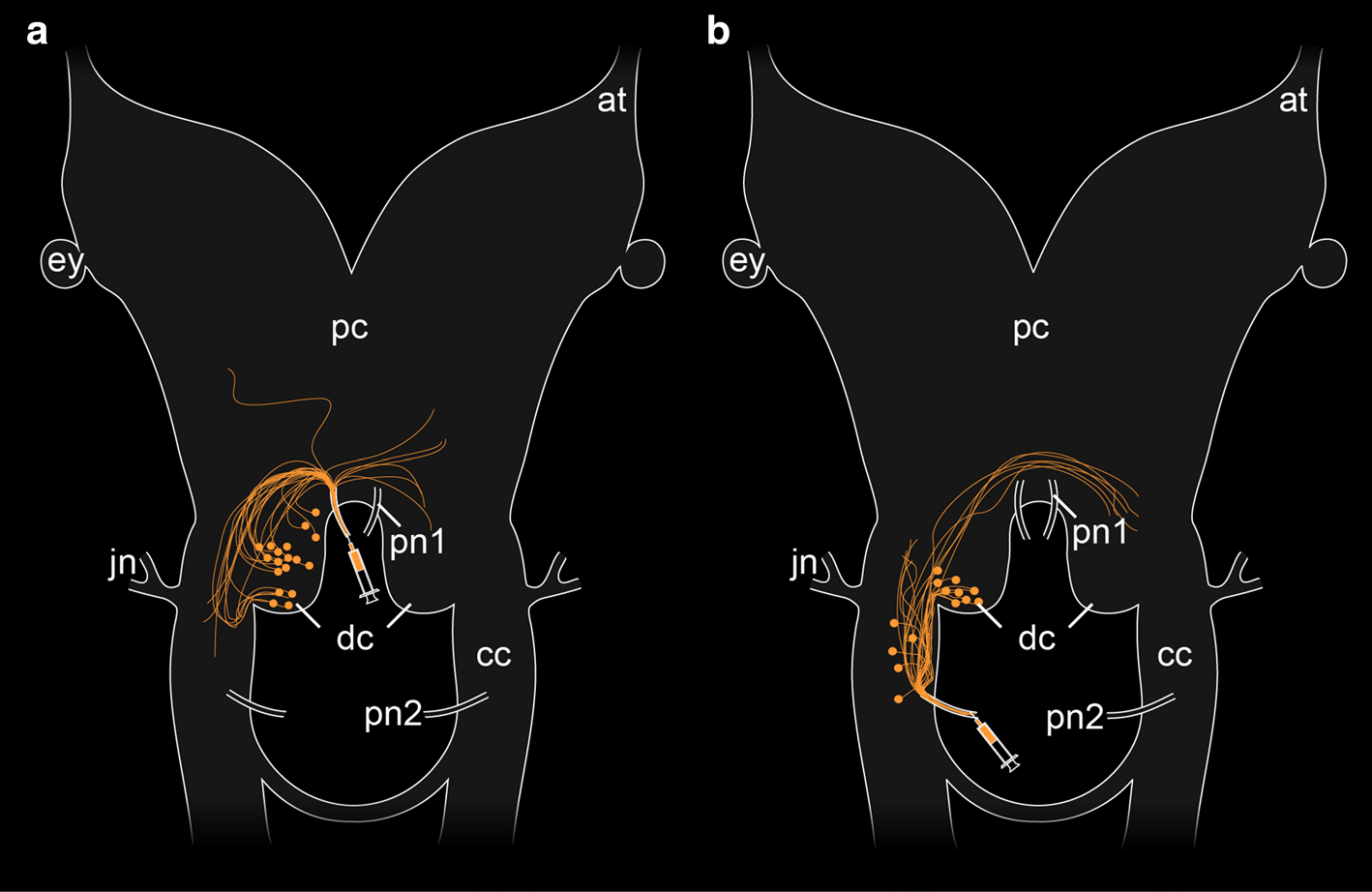
Simplified diagrams summarizing the results of retrograde fills of the pharyngeal nerves in *Euperipatoides rowelli*. Anterior is *up*. *Syringes* point to the corresponding fill sites. Note the somata associated with the anterior and posterior pharyngeal nerves that are situated within the deutocerebrum (**a**, **b**) and additional somata associated with the posterior pharyngeal nerve that are located in the connecting cord (**b**). *at* antennal tract, *cc* connecting cord, *dc* deutocerebrum, *ey* eye, *jn* jaw nerve, *pc* protocerebrum, *pn1* anterior pharyngeal nerve, *pn2* posterior pharyngeal nerve

Our retrograde fills of the anterior and posterior pairs of pharyngeal nerves in *E. rowelli* revealed that the majority of neuronal somata supplying the onychophoran pharynx are associated with the deutocerebrum (Fig. 7a, b). This innervation pattern of the adult pharynx corresponds to the embryonic origin of this structure from the walls of the stomodeum, which arise from the invaginating ectoderm near the border between the first and second body segments [15, 21] (cf. Fig. 1b). Since the stomodeum arises posterior to the *hedgehog* domain of the antennal segment but anterior to that of the jaw segment [12], it can be unambiguously assigned to the second, i.e., deutocerebral body segment. Thus, the segmental identity of the pharynx and its anlage as a deutocerebral structure is supported by both previous developmental and our neuroanatomical data. Besides the pharynx, additional structures involved in feeding that are innervated by the deutocerebrum are the jaws and several lateral or posterolateral lip papillae surrounding the mouth [8, 14]. This suggests that the deutocerebrum plays a role in coordinating feeding structures in Onychophora.

### Segmental identity of post-oral commissures in Onychophora

The median commissures that link the two widely separated nerve cords of onychophorans do not show any obvious segmental arrangement but are instead organized in a ladder-like fashion along the body (reviewed by Whitington and Mayer [16] and Mayer [7]). Using the position of leg nerves as segmental landmarks, previous studies have shown that the number of median commissures varies between 8 and 10 per segment [16–19, 27]. This variation in number and the lack of segmental ganglia in onychophorans generally render it difficult to assign each median commissure to a corresponding body segment.

**Fig. 8:**
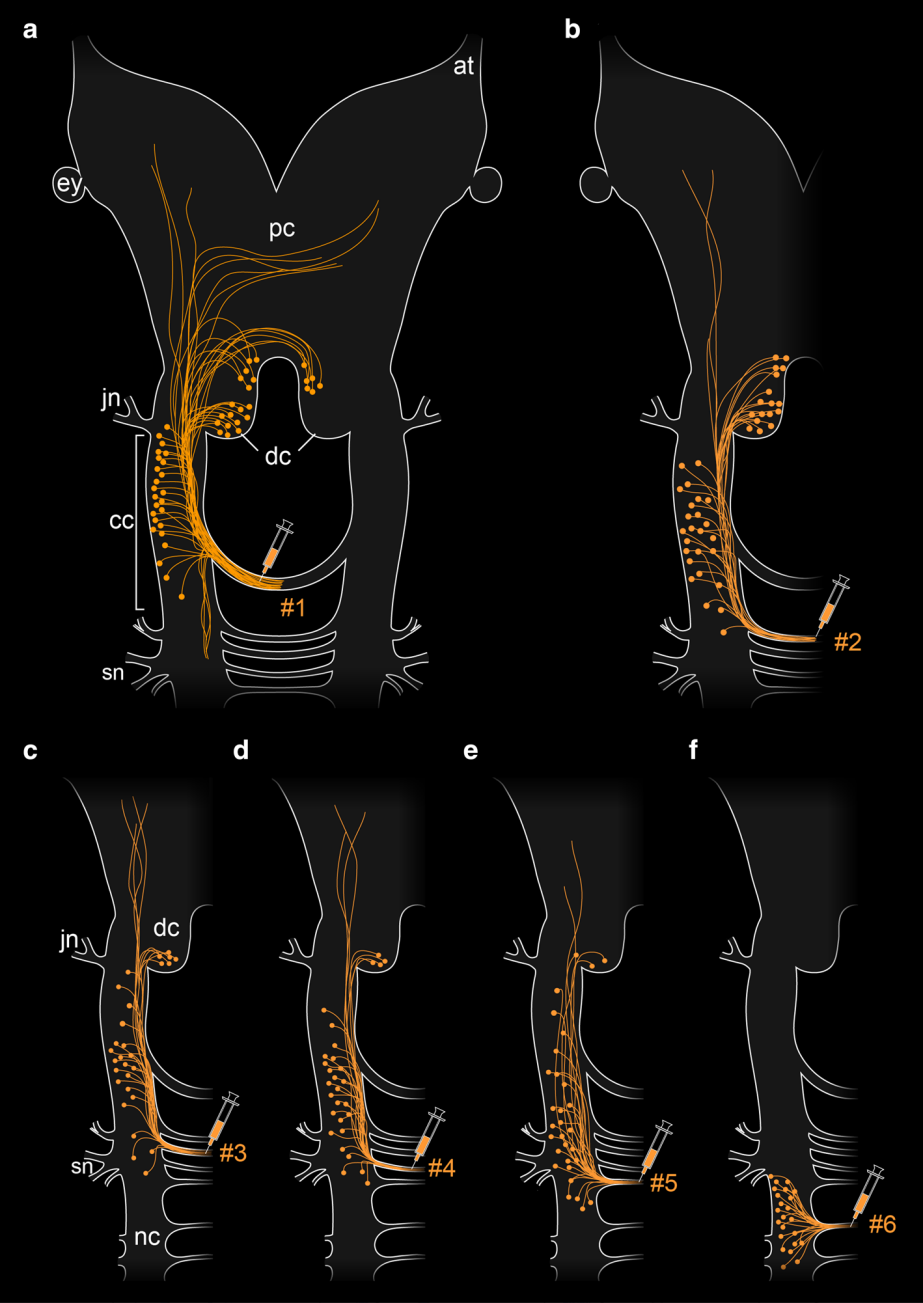
Simplified diagrams summarizing the results of retrograde fills of commissures #1 to #6 in *Euperipatoides rowelli*. Anterior is *up*. *Syringes* point to the corresponding fill sites. **a–f ** Note the diminishing number of somata within the deutocerebrum and a decreasing shift in position of somata within the connecting cords from anterior to posterior (commissure #6 is the first post-oral commissure that shows no anterior shift in position of the associated somata). *at* antennal tract, *cc* connecting cord, *dc* deutocerebrum, *ey* eye, *jn* jaw nerve, *nc* ventral nerve cord, *pc* protocerebrum, *sn* slime papilla nerves

**Fig. 9:**
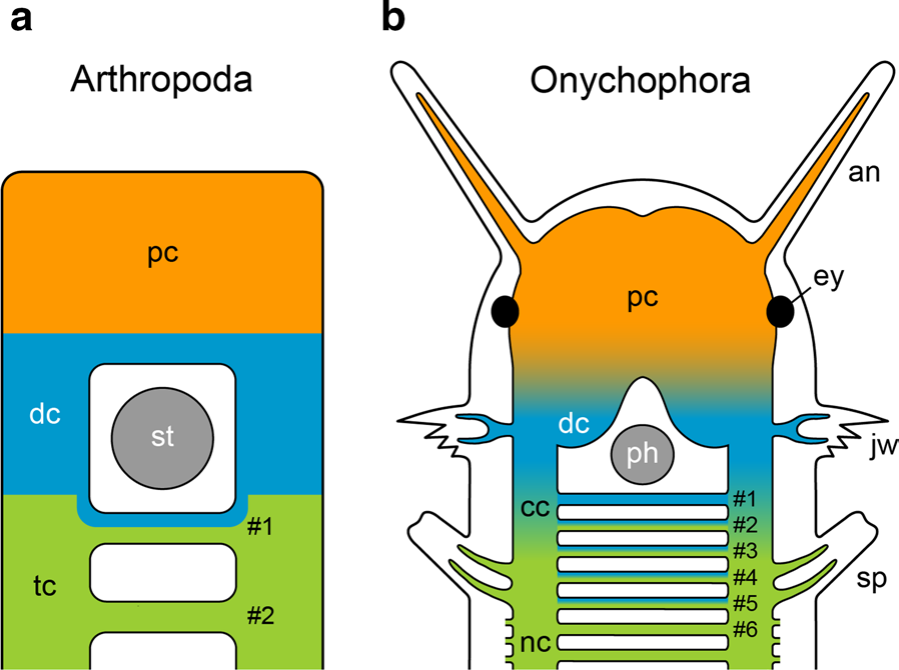
Segmental organization of the brain and composition of anteriormost post-oral commissures in arthropods and onychophorans. Anterior is *up*. Post-oral commissures are numbered. **a** Condition in arthropods (modified from Fanenbruck et al. [1]). Note that the first post-oral commissure contains both deutocerebral (*blue*) and tritocerebral fibers (*green*). **b** Situation in Onychophora, as revealed in the present study. The first post-oral commissure is most likely associated with the deutocerebrum, whereas commissures #2 to #5 consist of both deutocerebral fibers (*blue*) and fibers associated with the anterior portion of nerve cords belonging to the slime papilla segment (*green*). *an* antenna, *cc* connecting cord, *dc* deutocerebrum, *ey* eye, *nc* nerve cord, *pc* protocerebrum, *ph* pharynx, *jw* jaw, *sp* slime papilla, *st* stomodeum, *tc* tritocerebrum

Our fills of trunk commissures revealed that their supplying neurons are grouped around each commissural basis. This holds true for all median commissures of the trunk, including commissure #6 (Fig. 8f). In contrast, post-oral commissures #1 to #5 display numerous anteriorly shifted somata, some of which clearly lie within the deutocerebrum (Fig. 8a–e). The anteriorly shifted position of somata makes it difficult to assign each post-oral commissure to a particular head segment. However, the position of somata within the deutocerebrum as well as in portions of nerve cords supplying the slime papillae suggests that commissures #2 to #5 receive fibers from both the second (jaw) and the third (slime papilla) segments (Fig. 8b–e). This pattern resembles the innervation of the first post-oral commissure in various arthropods, which contains both deutocerebral and tritocerebral fibers [1, 22–26] (Fig. 9a, b).

Our neuroanatomical data from *E. rowelli* further revealed that the first post-oral commissure differs from commissures #2 to #5 in that it is thicker and longer and lies relatively further anteriorly, as it is associated with the connecting cords rather than with the anterior portions of nerve cords supplying the slime papillae (cf. Fig. 2a, b). Moreover, the first post-oral commissure is the only commissure with somata in the contralateral brain hemispheres relative to the fill site (Figs. 3b, 8a). Despite our detailed data on the position of supplying neurons, the first post-oral commissure of onychophorans is difficult to assign to a particular head segment, as numerous associated somata are located in the connecting cords, the segmental identity of which is unknown. However, since most commissural somata are situated in the anterior portions of the connecting cords as well as in the deutocerebrum and none of them is associated with the regions of nerve cords innervating the slime papillae, we suggest that the first post-oral commissure mainly, if not exclusively, receives fibers from neurons that belong to the second (jaw) segment (Fig. 9b). Clarifying the segmental identity and embryonic origin of the connecting cords will be crucial for substantiating this hypothesis.

In summary, our findings suggest that the first post-oral commissure of onychophorans is most likely associated with the second (deutocerebral) part of the brain supplying the jaws [8]. Interestingly, Henry [20] reported a close association of the first post-oral commissure with the ventral pharyngeal wall, which was also evident in our preparations of the nervous system in *E. rowelli*. We therefore cannot exclude that at least some commissural fibers might project into the pharynx. If true, this would imply that the first post-oral commissure is involved in the control of pharyngeal function, i.e., it might play a role in controlling the ingestion of food.

## Conclusion: implications for the evolution of the onychophoran brain

The overall organization of the onychophoran brain differs from that of arthropods in that it has only two segmental regions, the protocerebrum and the deutocerebrum [8] (Fig. 1a). In contrast, representatives of arthropods typically have at least one additional segmental brain region, the tritocerebrum (e.g. [1–3, 6, 28]; but note also that branchiopod crustaceans have a bipartite brain since the tritocerebrum is spatially separated from the proto- and deutocerebrum [29]). The typical arthropod brain is a compound structure, which is formed by the morphological fusion of separate embryonic ganglion anlagen [5]. Thus, the ontogeny of the arthropod brain might recapitulate evolutionary changes that have taken place in the arthropod lineage, as the tripartite brain of arthropods might have evolved from separate ganglia that have fused to form a syncerebrum [5] (Additional file 1). A segmentally ganglionated nervous system is also present in tardigrades [30–34], one of the closest relatives of arthropods. However, developmental studies [35] argue against a multisegmented brain in tardigrades. Additionally, the position of the stomatogastric ganglion in the second trunk segment [4] suggests that this segment in tardigrades is homologous to the arthropod tritocerebrum. Accordingly, the ganglion of the first trunk segment corresponds to the arthropod deutocerebrum and therefore the tardigrade brain consists of only a single segmental region corresponding to the arthropod protocerebrum. Thus, Tardigrada might represent the ancestral “rope ladder-like” condition, i.e. one ganglion per segment, suggesting that ganglionization occurred before the fusion of brain neuromeres, as is found in arthropods. Such a ganglionic fusion is unlikely to have occurred in onychophorans because their last common ancestor most likely did not possess any segmental ganglia and because no segmental ganglion anlagen exist in the onychophoran embryo [17–19]. Hence, the bipartite brain of onychophorans might have evolved by an entirely different process.

Our retrograde fills revealed a graded arrangement of anteriorly shifted somata associated with post-oral commissures #1 to #5 in *E. rowelli* (cf. Fig. 8a–e). The number of deutocerebral somata associated with each commissure decreases posteriorly, i.e., commissure #1 shows the highest and commissure #5 the lowest numbers, whereas none of the subsequent commissures, beginning with commissure #6, exhibits somata located in the deutocerebrum. Based on this graded arrangement of commissural neurons, we assume that the deutocerebral somata might have migrated anteriorly along each nerve cord, i.e., towards the protocerebrum, in the onychophoran lineage (Additional file 2). The medullary organization of the ventral nervous system in onychophorans [7, 16–19, 36] might have allowed the neuronal somata to change their position along the antero-posterior body axis in their last common ancestor. This hypothesis does not require an assumption of pre-existing segmental ganglia in the onychophoran ancestor that might have fused to a compound brain, as in arthropods.

These findings imply that the bipartite syncerebrum of onychophorans and the tripartite brain of arthropods might have evolved convergently by two different processes: an anterior migration of neurons in the onychophoran lineage, and a fusion of pre-existing segmental ganglia in the arthropod lineage (Additional files 1, 2). To distinguish between the two types of brain, Richter et al. [5] suggested the terms “compound” for the arthropod brain and “composite” for the onychophoran brain—a suggestion that receives support from our observations.

**Fig. 10:**
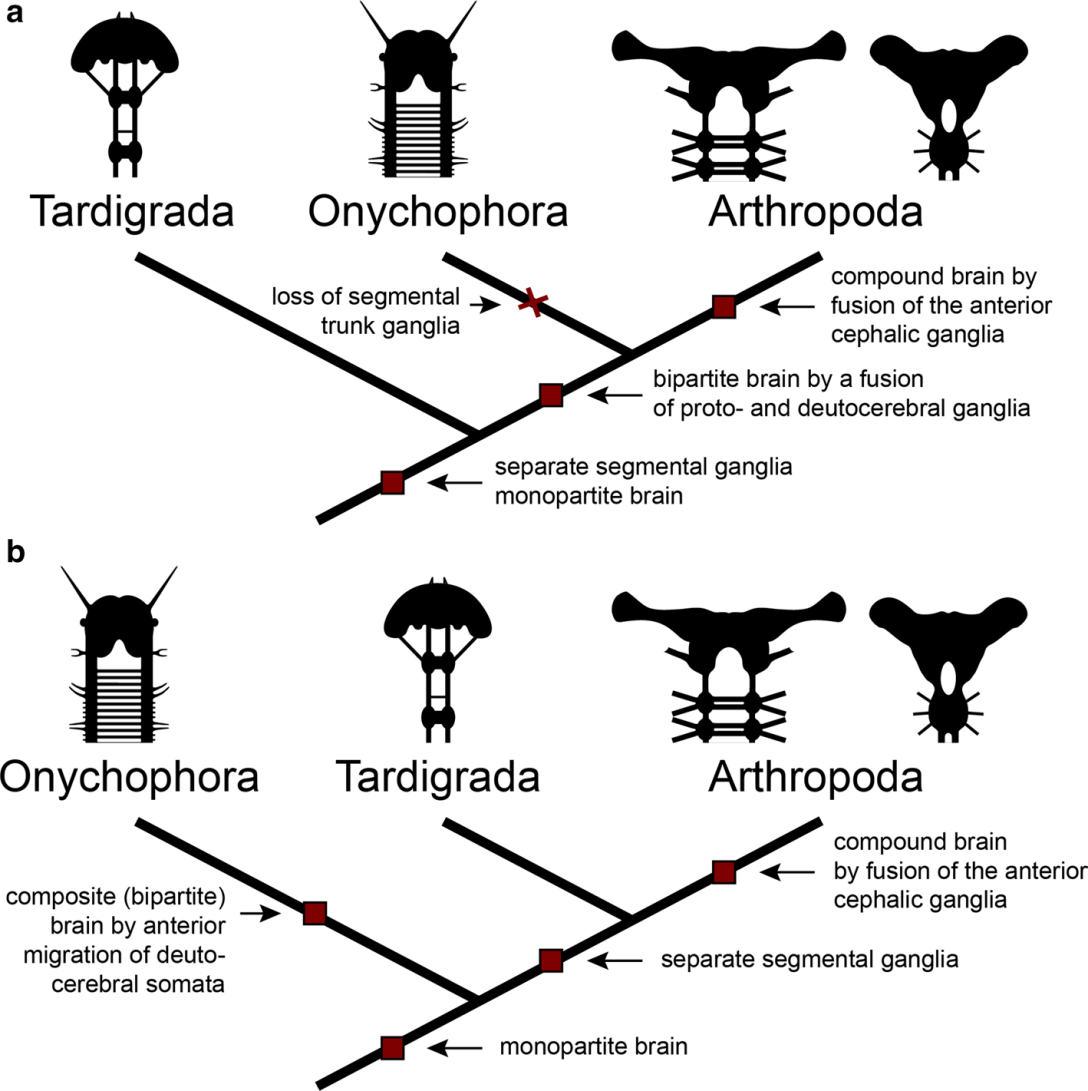
Alternative hypotheses of the evolution of the panarthropod brain. Scenario of the evolution of the brain suggesting that either **a** segmental ganglia or **b** medullary nerve cords were the ancestral feature in panarthropods

Within the group comprising onychophorans, tardigrades and arthropods (panarthropods) the position of tardigrades and onychophorans is still unresolved [31, 37–40]. Onychophorans are regarded as the sister group to either arthropods (Fig. 10a) or arthropods and tardigrades (Fig. 10b). Assuming that tardigrades form the sister group of onychophorans and arthropods (Fig. 10a), the last common ancestor of panarthropods might have possessed separate segmental ganglia and a monopartite brain. After a fusion of the two anteriormost ganglia to a bipartite brain in the last common ancestor of onychophorans and arthropods, the remaining segmental ganglia were lost within the onychophoran lineage, whereas most arthropods evolved a tripartite brain due to an additional fusion (Fig. 10a). Consequently, this scenario assumes that both onychophorans and arthropods possess a compound brain that evolved in their last common ancestor.

Alternatively, assuming that Onychophora form the sister group to tardigrades and arthropods, the last common ancestor of panarthropods might have possessed a monopartite brain and medullary ventral nerve cords (Fig. 10b). Within the onychophoran lineage, neurons of the second body segment migrated towards the protocerebrum and gave rise to a bipartite, composite brain. The last common ancestor of tardigrades and arthropods, on the other hand, evolved separate ganglia. The three anteriormost ganglia fused within the arthropod lineage, thus forming the arthropod compound brain (Fig. 10b).

As neither of the two onychophoran groups (Peripatidae nor Peripatopsidae) show any evidence of a loss of ganglia [17, 18], we favor the second scenario over the first one. Furthermore, our presented findings support the hypothesis that the onychophoran brain evolved by an anterior migration of deutocerebral somata rather than the fusion of two initially separate ganglia.

## Methods

### Specimen collection and maintenance

Specimens of *Euperipatoides rowelli* Reid, 1996 (Onychophora, Peripatopsidae) were collected from rotted logs in the Tallaganda State Forest (New South Wales, Australia; 35°26′S, 149°33′E, 954 m) in January 2013. Permission for specimen collection was obtained from the Forestry Commission of New South Wales (permit no. SPPR0008). The animals were kept in plastic jars (diameter 55 mm, height 70 mm) with perforated lids at 18 °C as described previously [41] and fed with decapitated crickets every 4 weeks.

### Retrograde fills of selected nerves

For neuronal staining, adult specimens were anesthetized in chloroform vapor for 10–25 s and cut open longitudinally along the dorsal side using fine scissors as described previously [14]. The brains with the nerve cords were dissected in physiological saline [42] and pinned down with tungsten needles in small Petri dishes coated with Sylgard^®^ (184 Silicone Elastomer Kit, DowCorning GmbH, Wiesbaden, Germany). Selected commissures that were cut at the midline and nerves (Table 1) were dissected from other tissue and a well of Vaseline was built around each isolated nerve, after which the saline was removed from the well and replaced with distilled water. A few crystals of dextran coupled to either tetramethylrhodamine or fluorescein (MW3000, lysine-fixable; Molecular Probes, Eugene, USA) were then added to the well filled with distilled water [43]. The preparations were incubated in the dark for 12–16 h at 4 °C, after which the well containing dextran was removed and the tissue fixed in 4 % paraformaldehyde in phosphate-buffered saline (PBS; 0.1 M, pH 7.4) for 2 h at 4 °C. After several rinses in PBS and dehydration through an ethanol series (50, 70, 90, 95 %, 2 × 100 %; 10 min each) the preparations were cleared in methyl salicylate and mounted between two coverslips.

**Table 1:** Number of fills performed for each commissure and pharyngeal nerve in specimens of *Euperipatoides rowelli*

Filled nerve	Number of fills
Commissure #1	10
Commissure #2	8
Commissure #3	7
Commissure #4	5
Commissure #5	5
Commissure #6	2
Trunk commissures	2
Anterior pharyngeal nerve	3
Posterior pharyngeal nerve	5

### Confocal microscopy, light microscopy and image processing

Whole-mount preparations of the central nervous system were analyzed with a fluorescence microscope (Leica Leitz DMR; Leica Microsystems, Wetzlar, Germany) and a confocal laser-scanning microscope (Leica TCS STED; Leica Microsystems). Confocal image stacks were processed with Leica ASAF v2.3.5 (Leica Microsystems), IMARIS 7.2.1 (Bitplane, Zurich, Switzerland), and ImageJ 1.48v (National Institutes of Health, Bethesda, MD, USA). Final panels and diagrams were designed using Adobe (San Jose, CA, USA) Photoshop CS5 and Illustrator CS5. Animations were designed using Adobe Flash CS5.

## Additional files

Additional file 1: Scenario on the evolution of the arthropod brain. Animation, swf file. Anterior is *up*. The compound brain of arthropods (sensu Richter et al. [5]) most likely evolved by a fusion of initially separate proto-, deuto- and tritocerebral ganglia. Note that the tripartite brain of arthropods alternatively might have evolved several times independently, as some groups, including branchiopod crustaceans, show a bipartite brain (e.g. [29]). Additional file 2: Scenario on the evolution of the onychophoran brain. Animation, swf file. Anterior is *up*. The composite brain of onychophorans (sensu Richter et al. [5]) most likely evolved by anteriorly moving somata, which give rise to the deutocerebrum.
